# Editorial: Pathogenic roles of T cells in autoimmunity

**DOI:** 10.3389/fimmu.2024.1401459

**Published:** 2024-04-03

**Authors:** Jinfang Xia, Jifeng Tang, Qiong Fu, Jinpiao Lin

**Affiliations:** ^1^ Department of Laboratory Medicine, Tongren Hospital, Shanghai Jiao Tong University School of Medicine, Shanghai, China; ^2^ Department of Rheumatology, Renji Hospital, School of Medicine, Shanghai Jiao Tong University, Shanghai, China

**Keywords:** autoimmunity, heterogeneity, pathogenicity, autoimmune diseases, helper T

In the immune system, many tolerance checkpoints exist to prevent self-antigens from stimulating the growth of self-reactive T and B lymphocytes. Moreover, thymic selection ensures that the repertoire of available T cells is both MHC-restricted and self-tolerant. However, these tolerance mechanisms are imperfect. When the immune system turns its antimicrobial defences upon normal components of the body, autoimmune diseases arise, which cause abnormal responses of B and T cells (1, 2). Helper T cells (Th), especially CD4 T cells play a crucial role in the pathogenesis of autoimmune diseases. Upon antigen peptide recognition via the T cell receptor (TCR), naive CD4 T cells undergo clonal expansion and differentiation into functionally distinct effector helper T (Th) cell subsets, including Th1, Th2, Treg, Th17 and Tfh cells. Th1 cells produce IFN-γ and express T-bet; Th2 cells produce IL-4 and IL-13 and express GATA3; Th17 cells produce IL-17 and IL-22 and express RORγt; and Tfh cells produce IL-21 and express BCL6. Treg cells that are induced in the periphery from naive precursors produce TGF-β and express Foxp3. Other T cell subsets include Th22 cells, which produce IL-22 and express the aryl hydrocarbon receptor (AHR) (3–5), and Th9 cells, which produce IL-9 and express the transcription factor PU.1 (6, 7). However, due to the heterogeneity and plasticity of Th cells, various over-activated T cell subpopulations like iTh1 (inflammatory Th1), iTh2, iTh17, etc. can express different cell phenotypes and play pathogenic roles in autoimmune diseases, which may be the potential cause of poor therapeutic outcomes for certain patients (8, 9). Therefore, exploring the exact reasons why such T cells become more pathogenic and targeting pathogenic T cells rather than all T cells, maybe more helpful in both treating and preventing autoimmune diseases from progressing.

In this Research Topic, titled “Pathogenic Roles of T Cells in Autoimmunity”, we aim to explore the knowledge regarding the origins, phenotypic characteristics, pathogenic mechanisms, and diagnostic value of pathogenic T cells in autoimmune diseases. We present a collection of papers exploring pathogenic roles of T Cells in various aspects of autoimmune disease, including vitiligo, idiopathic inflammatory myopathies (IIMs) and primary Sjogren Syndrome (pSS).

Vitiligo is an autoimmune skin disease. CD8+ T cells play a crucial role in the pathogenesis of vitiligo. Leptin is mainly synthesized and secreted by white adipose tissue, which is critical for metabolic homeostasis and can affect the differentiation and function of T cells. The effect of leptin on vitiligo remains unclear. Wu et al. reported that leptin could promote the progression of vitiligo by enhancing the production of interferon-γ and granzyme B in CD8+ T cells, suggesting that leptin may become a new target for vitiligo treatment.

Idiopathic inflammatory myopathies (IIM) is characterized by subacute proximal muscle weakness. T cells of different lineages have been implicated in the pathogenesis of IIM. Anang et al. had shown the presence of highly expanded TCRβ clones in muscle tissues and peripheral blood of IIM patients. Moreover, increased clonal expansion and decreased diversity of the TCRβ repertoire in muscle tissue correlated with increased disease activity, which supported a role for specific clonal T cell responses in muscle tissue in the pathogenesis of the IIM.

Primary Sjogren’s Syndrome (pSS) is an autoimmune disease characterized by immune cells infiltration, including T cells, B cells, plasma cells and macrophages. T follicular helper (Tfh) cells is a distinct Th lineage specialized in supporting B cell maturation and survival in germinal centers. However, research on the distribution and expression of Tfh cells in pSS is rare. By transcriptome analysis, Luo et al. revealed that The presence of Tfh cells (CD4+CXCR5+ICOS+) in germinal centers (GC) and the labial glands of pSS patients may play a crucial role in the pathogenesis of pSS.

γδT cells play crucial roles in infection and autoimmune diseases partly through their ability to secrete IL-17. Murine IL-17-producing γδT (γδT17) cells are divided into two subsets: natural γδT17 (nγδT17) cells and inducible γδT17 cells. IL-21, which is involved in the differentiation of Th17 cells, in the development and pathophysiology of γδT17 cells remain unknown. Ishikawa et al. have shown that IL-21 is dispensable for the foetal thymic natural γδT17 development of cells but is required for the peripheral maintenance of Vγ4+ natural γδT17 cells, adding new insight into the mechanisms of IL-21-mediated pathogenesis of autoimmune diseases.

In addition, this Research Topic features one review. Rheumatoid arthritis (RA) and postmenopausal osteoporosis (PMOP) are common bone-immune diseases. The imbalance between T helper 17 (Th17) and regulatory T cells (Tregs) plays a key regulatory role in bone remodelling disorders in RA and PMOP. Wang et al. have summarized the potential roles of Th17, Treg, and Th17/Treg imbalance in regulating bone remodelling in RA and PMOP. The maintenance of Th17/Treg balance could be considered as a therapeutic strategy for the treatment of RA and PMOP.

There are still many shortages in this topic that need to be improved. Because the researchers have the different academic backgrounds, each of the above studies can be considered as an independent research. However, these studies provide insights into the impact of pathogenic roles of T cells in autoimmune diseases. This topic will attract the attention of researchers in related fields, including pathogenesis, therapeutic strategies and diagnostic of autoimmune diseases and T cells differentiation. We hope that these findings will promote the publication of original findings and provide novel insights in the autoimmunity research field.

## Author contributions

JX: Writing – original draft. JT: Writing – review & editing. QF: Writing – review & editing. JL: Writing – review & editing, Conceptualization.
